# Publisher’s Note: Withdraw and Republication of “UV-Induced Radical Photo-Polymerization: A Smart Tool for Preparing Polymer Electrolyte Membranes for Energy Storage Devices”

**DOI:** 10.3390/membranes2040705

**Published:** 2012-10-17

**Authors:** Shu-Kun Lin

**Affiliations:** MDPI AG, Postfach, CH-4005 Basel, Switzerland; E-Mail: lin@mdpi.com

It has been brought to the corresponding author’s attention by the administration office that some of the authors present in this paper [1] are contradicting with the rules and regulation of some of the confidential industrial projects which have been signed with strict regulations. Now it has aroused as a big trouble and, consequently, to solve this problem all the authors have determined that it should be retracted. This decision has been taken purely for bureaucratic aspect. We apologize for any inconvenience this may cause.

The new version is published as [2]. 

## References

[1] Nair J.R., Chiappone A., Destro M., Jabbour L., Zeng J., Di Lupo F., Garino N., Meligrana G., Francia C., Gerbaldi C. (2012). UV-Induced Radical Photo-Polymerization: A Smart Tool for Preparing Polymer Electrolyte Membranes for Energy Storage Devices. Membranes.

[2] Nair J.R., Chiappone A., Destro M., Jabbour L., Meligrana G., Gerbaldi C. (2012). UV-Induced Radical Photo-Polymerization: A Smart Tool for Preparing Polymer Electrolyte Membranes for Energy Storage Devices. Membranes.

